# Exercise therapy facilitates neural remodeling and functional recovery post-spinal cord injury via PKA/CREB signaling pathway modulation in rats

**DOI:** 10.1093/burnst/tkae058

**Published:** 2025-01-22

**Authors:** Xinwang Ying, Qingfeng Xie, Yanfang Zhao, Jiamen Shen, Junqing Huang, Zhiyi Feng, Liuxi Chu, Junpeng Xu, Dawei Jiang, Ping Wu, Yanming Zuo, Shengcun Li, Chang Jiang, Xiaokun Li, Zhouguang Wang

**Affiliations:** The Orthopaedic Center, The Affiliated Wenling Hospital of Wenzhou Medical University (The First People’s Hospital of Wenling), 333 Chuanan Road, Chengxi Street, Wenling City, Zhejiang Province 317500, China; Department of Physical Medicine and Rehabilitation, The Second Affiliated Hospital and Yuying Children's Hospital of Wenzhou Medical University, 109 Xueyuan West Road, Lucheng District, Wenzhou City, Zhejiang Province 325000, China; National Key Laboratory of Macromolecular Drug Development and Manufacturing, School of Pharmaceutical Science, Wenzhou Medical University, Zhongxin North Road, Chashan Higher Education Park, Ouhai District, Wenzhou City, Zhejiang Province 325035, China; Department of Physical Medicine and Rehabilitation, The Second Affiliated Hospital and Yuying Children's Hospital of Wenzhou Medical University, 109 Xueyuan West Road, Lucheng District, Wenzhou City, Zhejiang Province 325000, China; National Key Laboratory of Macromolecular Drug Development and Manufacturing, School of Pharmaceutical Science, Wenzhou Medical University, Zhongxin North Road, Chashan Higher Education Park, Ouhai District, Wenzhou City, Zhejiang Province 325035, China; National Key Laboratory of Macromolecular Drug Development and Manufacturing, School of Pharmaceutical Science, Wenzhou Medical University, Zhongxin North Road, Chashan Higher Education Park, Ouhai District, Wenzhou City, Zhejiang Province 325035, China; Oujiang Laboratory (Zhejiang Lab for Regenerative Medicine, Vision and Brain Health), School of Pharmaceutical Science, Wenzhou Medical University, No. 999 Jinshi Road, Yongzhong Street, Longwan District, Wenzhou City, Zhejiang Province 325000, China; National Key Laboratory of Macromolecular Drug Development and Manufacturing, School of Pharmaceutical Science, Wenzhou Medical University, Zhongxin North Road, Chashan Higher Education Park, Ouhai District, Wenzhou City, Zhejiang Province 325035, China; Oujiang Laboratory (Zhejiang Lab for Regenerative Medicine, Vision and Brain Health), School of Pharmaceutical Science, Wenzhou Medical University, No. 999 Jinshi Road, Yongzhong Street, Longwan District, Wenzhou City, Zhejiang Province 325000, China; Wenzhou Medical University, Affiliated Cixi Hospital, No. 999, South Second Ring Road East, Hushan Street, Cixi City, Ningbo City, Zhejiang Province 315300, China; Wenzhou Medical University, Affiliated Cixi Hospital, No. 999, South Second Ring Road East, Hushan Street, Cixi City, Ningbo City, Zhejiang Province 315300, China; National Key Laboratory of Macromolecular Drug Development and Manufacturing, School of Pharmaceutical Science, Wenzhou Medical University, Zhongxin North Road, Chashan Higher Education Park, Ouhai District, Wenzhou City, Zhejiang Province 325035, China; National Key Laboratory of Macromolecular Drug Development and Manufacturing, School of Pharmaceutical Science, Wenzhou Medical University, Zhongxin North Road, Chashan Higher Education Park, Ouhai District, Wenzhou City, Zhejiang Province 325035, China; Department of Physical Medicine and Rehabilitation, The Second Affiliated Hospital and Yuying Children's Hospital of Wenzhou Medical University, 109 Xueyuan West Road, Lucheng District, Wenzhou City, Zhejiang Province 325000, China; The Orthopaedic Center, The Affiliated Wenling Hospital of Wenzhou Medical University (The First People’s Hospital of Wenling), 333 Chuanan Road, Chengxi Street, Wenling City, Zhejiang Province 317500, China; Oujiang Laboratory (Zhejiang Lab for Regenerative Medicine, Vision and Brain Health), School of Pharmaceutical Science, Wenzhou Medical University, No. 999 Jinshi Road, Yongzhong Street, Longwan District, Wenzhou City, Zhejiang Province 325000, China; The Orthopaedic Center, The Affiliated Wenling Hospital of Wenzhou Medical University (The First People’s Hospital of Wenling), 333 Chuanan Road, Chengxi Street, Wenling City, Zhejiang Province 317500, China; Oujiang Laboratory (Zhejiang Lab for Regenerative Medicine, Vision and Brain Health), School of Pharmaceutical Science, Wenzhou Medical University, No. 999 Jinshi Road, Yongzhong Street, Longwan District, Wenzhou City, Zhejiang Province 325000, China

**Keywords:** Spinal cord injury, Exercise therapy, PKA/CREB, Neural remodeling

## Abstract

**Background:**

Neuronal structure is disrupted after spinal cord injury (SCI), causing functional impairment. The effectiveness of exercise therapy (ET) in clinical settings for nerve remodeling post-SCI and its underlying mechanisms remain unclear. This study aims to explore the effects and related mechanisms of ET on nerve remodeling in SCI rats.

**Methods:**

We randomly assigned rats to various groups: sham-operated group, sham-operated + ET, SCI alone, SCI + H89, SCI + ET, and SCI + ET + H89. Techniques including motor-evoked potential (MEP), video capture and analysis, the Basso–Beattie–Bresnahan (BBB) scale, western blotting, transmission electron microscopy, hematoxylin and eosin staining, Nissl staining, glycine silver staining, immunofluorescence, and Golgi staining were utilized to assess signal conduction capabilities, neurological deficits, hindlimb performance, protein expression levels, neuron ultrastructure, and tissue morphology. H89—an inhibitor that targets the protein kinase A (PKA)/cAMP response element-binding (CREB) signaling pathway—was employed to investigate molecular mechanisms.

**Results:**

This study found that ET can reduce neuronal damage in rats with SCI, protect residual tissue, promote the remodeling of motor neurons, neurofilaments, dendrites/axons, synapses, and myelin sheaths, reorganize neural circuits, and promote motor function recovery. In terms of mechanism, ET mainly works by mediating the PKA/CREB signaling pathway in neurons.

**Conclusions:**

Our findings indicated that: (1) ET counteracted the H89-induced suppression of the PKA/CREB signaling pathway following SCI; (2) ET significantly alleviated neuronal injury and improved motor dysfunction; (3) ET facilitated neuronal regeneration by mediating the PKA/CREB signaling pathway; (4) ET enhanced synaptic and dendritic spine plasticity, as well as myelin sheath remodeling, post-SCI through the PKA/CREB signaling pathway.

## Graphical Abstract

**Graphical Abstract:**
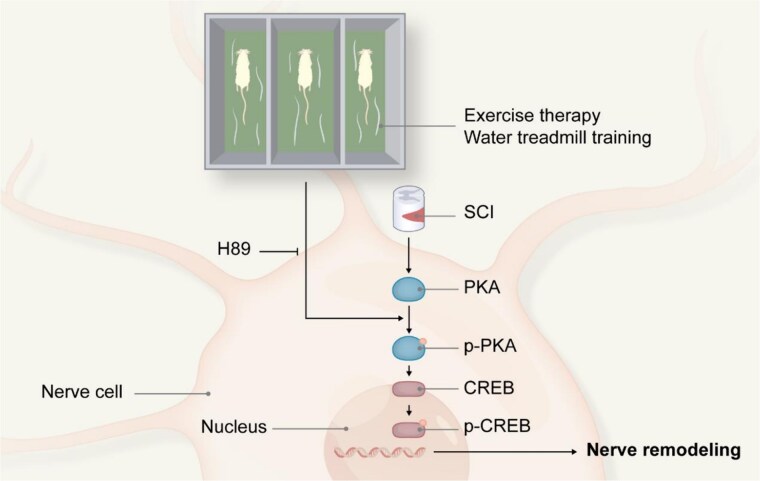

Highlights

- Exercise therapy counteracts the H89-induced suppression of PKA/CREB signaling pathway following SCI.
- Exercise therapy significantly alleviates neuronal injury and improves motor dysfunction
- Exercise therapy facilitates neuronal regeneration by mediating the PKA/CREB signaling pathway
- Exercise therapy enhances synaptic and dendritic spine plasticity, as well as myelin sheath remodeling post-SCI, through the PKA/CREB signaling pathway

## Background

The incidence of spinal cord injury (SCI) is increasing, and due to its great devastation, it brings a huge economic burden on both clinical and social systems [1, 2]. After SCI, neurons are seriously damaged, making it difficult for them to form effective structural or functional connections [3]. After SCI, axons will be damaged and demyelinated, some neurons in the injured area will be lost, and endogenous neurogenesis will be restricted [4]. Some studies have demonstrated that the ischemic and anoxic microenvironment resulting from SCI is not conducive to dendritic modification either, and the number of dendritic spines decreases accordingly, resulting in a weakening of the connection with neurons [5–7]. Furthermore, SCI weakens synaptic plasticity. Neuroplasticity refers to the capacity of the nervous system to undergo functional and anatomical changes in response to stimuli or injuries that occur during the learning process [8]. Currently, the drugs and surgeries used to treat the occurrence and development of spinal cord injuries focus on reducing complications and cannot provide effective fundamental treatment. Therefore, finding an efficient and safe intervention method is the current research direction.

Stimulation of extracellular signal-related kinase (ERK)/cAMP response element-binding (CREB) or protein kinase A (PKA)/CREB signaling pathways can promote the secretion of various neurotrophic factors [9, 10]. CREB acts on DNA and prompts the production of brain-derived neurotrophic factor, thereby playing an important role in brain development and neurogenesis [11]. Phosphorylation of CREB increases the excitability of rodent hippocampal and striatal neurons, while inhibition of CREB decreases neuronal excitability. Studies have confirmed that CREB is crucial for neuronal development during embryogenesis and is key to the regeneration of peripheral nervous system (PNS). It is also believed to contribute to the postnatal development and regeneration of the injured adult nervous systems [12]. CREB can be regulated by various kinases (such as PKA) and phosphatases and can have multiple functions, such as nerve growth, binding, development, and structure formation in nervous system gene expression [13, 14]. The PKA/CREB signaling pathway is crucial for integrating and regulating neuronal responses to external stimuli, positively influencing nerve cell survival, growth, and synaptic plasticity [15, 16].

As a rehabilitation strategy, exercise therapy (ET) has been widely used in patients with stroke or SCI because of its non-invasiveness and low cost. Through active or passive exercise, the motor function of patients can be restored. Given the lower limb paralysis that occurs after SCI in rats, both swimming and treadmill training were combined to reduce the resistance of rats during exercise and increase exposure to physical stimuli. In a previous study, we investigated vascular units after SCI. The main purpose of this study is to explore the effects of ET on neural remodeling and its related mechanisms.

## Methods

### Reagents and antibodies

Proteintech (IL, USA) provided us with microtubule-associated protein-2 (17490-1-AP) and Anti-synapsin I (20258-1-AP) antibodies. In addition, Abcam (MC, UK) supplied the antibodies for 5-bromo-20-deoxyuridine (BrdU) (ab6326), Postsynaptic Density Protein 95 (PSD95) (ab238135), and neurofilament-200(NF200) (ab215903). PKA alpha/beta/gamma CAT antibody (AF7746), phospho-PKA(p-PKA) alpha/beta/gamma CAT (Thr198) (AF7246), growth-associated protein-43 (GAP43) (AF6715), as well as glyceraldehyde-3-phosphatedehydrogenase (GAPDH) (AF7021) antibodies were supplied by Affinity (OH, USA). Novus (CO, USA) supplied neuron-specific nuclear protein (NeuN) (D3S3I). Additionally, anti-recombinant doublecortin (DCX) antibody (GB114319-100), anti-phospho-CREB (p-CREB) (S133) rabbit pAb (GB114322–100), and anti-CREB Rabbit pAb were supplied by Servicebio (Wuhan, China). H-89, (PKA inhibitor) (130964–39-5) was provided by GlpBio (CA, USA). An FD Rapid Golgi Stain Kit (PK401) was supplied by FD NeuroTechnologies, Inc. (MD, USA).

### Animals

In total, 200 adult male SD rats, each weighing between 200 and 250 g, were provided by Shanghai Laboratory Animal Centre. These rats underwent random assignment to six groups as follows: Sham-operated (*n* = 30; group S); Sham-operated + ET (*n* = 30; group SE); SCI (*n* = 35; group M); SCI + H89 (*n* = 35; group MI); SCI + ET (*n* = 35; group EM); SCI + ET + H89 (*n* = 35; group IEM). All experimental procedures received approval from the Animal Research Committee of Wenzhou Medical University (reference number wydw2020-0602). Such experiments were performed in strict adherence to the guidelines outlined in National Institutes of Health Guide for the Care and Use of Laboratory Animals.

### SCI model

The rats were maintained under anesthesia with 30 mg/kg of 2% pentobarbital sodium. After shaving the area between T9 and T11 on their backs, a 15-mm incision was done along the midline of the skin. The exposed site was subjected to impact using a New York University impactor configured at 10 g × 20 cm. Successful establishment of the SCI model in SCI groups was confirmed by the observation of body tremors, tail swaying, and lower limb retraction. The wound was subsequently sutured and disinfected with alcohol. To prevent urinary tract infections, the rats’ bladders were emptied, and their urethras were cleaned every morning and evening.

### Exercise therapy

The treadmill machine settings were consistent with our previous studies [17]. In addition, the temperature of the water was consistently preserved at 30°C. Starting from the second day after SCI, rat groups SE, EM, and IEM underwent ET treatment for 14 days. Before each experiment, placing the rats in water at 30°C was deployed to encourage their defecation. Following experiments, the rats underwent cleaning and drying before being returned to their cages.

### Functional behavior evaluation

At 1, 3, 7, and 14 day past injury (DPI) throughout the intervention, we used the Basso–Beattie–Bresnahan (BBB) scale to assess the recovery of hindlimb motor function in rats. Hindlimb behavioral assessments were conducted using a sophisticated method, following procedures from previous research [18, 19]. High-frame rate cameras were used to record the straight-line walking process of all experimental rats on the runway, and DeepLabCutGUI2.3.4 was used to mark, track, and analyze the trajectories of experimental rats. Use MatLab2022A to draw the stick view of the hindlimb and the angle of the hip joint, knee joint, and ankle joint. The hindlimb motion data of S, M, IM, EM, and IEM groups were calculated, and the angle differences between groups were compared using a scatter plot. Simultaneously, the motor-evoked potential (MEP) is carried out according to the following method [18, 20]. The recording electrode was placed into the gastrocnemius muscle on both sides of the leg, the stimulation electrode was placed at the upper end of the injury center, and the reference electrode was placed in the subcutaneous tissue of the rat abdomen. The spinal cord of rats was stimulated with an electrical stimulator (10 mA, 0.1 ms, 1 Hz) once every 15 s, and the amplitude of evoked potential after each stimulation was recorded. The above data were collected by two independent examiners without knowledge of treatment groups.

### Drug injection

To stain the rats with BrdU, 10 mg/ml of a 1% solution was administered via intraperitoneal injection at 4-h intervals, three doses in total, before the rats were euthanized [21].

Following SCI, the rats in IM and IEM groups received an intrathecal injection of the PKA inhibitor, H89 (3.5 μg/kg), once daily for a week, while an equal volume of saline was administered to the other groups [22–24].

### Tissue preparation

The rats in each group underwent sacrifice at 14 DPI following SCI. The T9–T11 segment tissues were rapidly excised, followed by 24-h storage in a similar fixative at 4°C. The tissues then underwent subsequent overnight immersion at 4°C in Phosphate buffered saline (PBS) with 20% and 30% concentrations. Afterward, the tissues were sectioned into 15 μm thick slices for further experiments.

### Hematoxylin and eosin staining and Nissl staining

Prepared slices (*n* = 5) underwent 3-min natural air-drying. Subsequently, the slices were stained using **hematoxylin and eosin** (H&E) staining, as well as Nissl staining (cresyl violet). Images were then captured using an Olympus BH-2 microscope (Olympus Optics, London, UK), and the Nissl bodies along with cavity area were quantitatively counted.

### Terminal deoxynucleotidyl transferase-mediated dUTP nick end labeling staining

The slices, once prepared (*n* = 5 in each group), underwent **terminal deoxynucleotidyl transferase**-mediated dUTP nick end labeling (TUNEL) staining. Permeabilization of the slides was carried out at room temperature for 10 min, followed by three washes, each lasting 15 min. Apoptotic cell detection was performed with the In Situ Cell Death Detection Kit (Roche Molecular Biochemicals). The images were examined with a BX51 microscope (Olympus), and apoptosis-positive cells were quantified with Image-Pro Plus 6.0 software.

### Glycine silver staining

Rat tissues from each group were made into paraffin sections. The sections were then washed using distilled water after applying xylene I and II for 20 min. Following this, the sections were placed in silver glycine stain solution C for 5 min, underwent three-time washes using distilled water, and were treated with silver glycine staining solution B for 3–5 min. After that, the sections were taken out, and the residual glycine silver stain solution B on tissues was quickly shaken off before placing them in the glycine silver stain solution A I (preheated at 45°C in advance). The reduction effect was observed at all times, and the slices were removed after a few seconds and quickly placed into silver glycine staining solution A II (preheated at 45°C in advance), and then promptly removed after a few seconds and washed with distilled water. If the background of the section was dark, it underwent quick treatment with glycine silver staining solution D for 1 s and then washed three times with distilled water. Anhydrous ethanol I, absolute ethanol II, and dehydrated ethanol III were applied, followed by 5-min immersions in xylene I and II for transparency. The slices were then sealed with neutral resin. The final steps included microscope examination, image acquisition, and analysis.

### Western blot analysis

After 14 days of intervention, 5 mm of spinal cord was taken from each group of rats. Typically, the tissue is thoroughly homogenized, and the supernatant is collected. Protein levels were subsequently measured with the bicinchoninic acid assay kit. After that, for electrophoresis, separation gels matched to the molecular weights of the target proteins were deployed. Following separation, the proteins were moved onto a polyvinylidene difluoride membrane (Bio-Rad, Hercules, CA, USA). Following a 5% milk closure for 90 min, the membrane underwent three-time washes using Tris-buffered saline solution/Tween (TBST), followed by incubation with a list of primary antibodies (4°C/16–24 h): p-PKA (1:1000), PKA (1:1000), p-CREB (1:500), CREB (1:1000), NF200 (1:1000), GAP43 (1:1000), MAP-2 (1:1000), Synapsin I (1:1000), PSD95 (1:1000), and GAPDH (1:1000). After incubation, the membrane was treated with secondary antibodies for 2 h, followed by three-time washing using TBST. The images underwent visualization using ChemiDicTM XRS plus Imaging System (Bio-Rad).

### Immunofluorescence staining

The sections, once prepared, underwent natural air-drying, three-time washes (0.01 M PBS), and room temperature blockage using 10% normal goat serum for 1 h. Subsequently, they underwent overnight incubation at 4°C with the following primary antibodies: rabbit anti-GAP43 (1:200), mouse anti-NF200 (1:200), mouse anti-Brdu (1:200), goat (rabbit) anti-NeuN (1:100), rabbit anti-p-PKA (1:500), rabbit anti-p-CREB (1:500), and rabbit anti-DCX (1:300). The next day, the sections underwent ambient temperature incubation with the corresponding secondary antibodies for 50 min. Afterward, counterstaining of the nuclei was done using 4,6-diamidino-2-phenylindole (DAPI). For visualization and analysis, for each animal, five fields from each of the three slides underwent random selection and analyses by ImageJ software (National Institutes of Health, Bethesda, MD, USA).

### Transmission electron microscopy

Fresh spinal cord tissues underwent isolation from the vertebrae with no prior heart perfusion. Subsequently, the tissues were rapidly cut into 1 mm^3^ size in addition to soaking in 2.5% glutaraldehyde, followed by three washes in PBS, each lasting 15 min. Afterward, the tissues were fixed in 1% osmium tetroxide for 1 h and subsequently stained with 1% uranyl acetate for 2 h. The tissues were then dehydrated using a series of gradient acetone solutions and embedded for coronal sectioning. The location was confirmed through semi-thin sectioning as well as toluidine blue staining, after which the tissues were cut into ultrathin sections. Finally, a Hitachi **transmission electron microscopy (**TEM) was used to examine the sections.

### Golgi staining

In brief, fresh tissue was placed in a preprepared solution (A:B was 1:1) and kept in a dark room at ambient temperature for 14 days. After transferring them to an impregnation solution C and keeping them in darkness (48 h/4°C), the samples were sliced into 150 μm thick and stained using standard protocols. Dendritic spine densities in T9–T10 neurons within the anterior horn were analyzed, with each cell being traced at 400× magnification.

### Statistical analysis

The experimental values are all expressed as the mean ± standard deviation (SD). Typically, the Shapiro–Wilk test was employed to assess the normality of the data [25], where *P*>.05 demonstrates a normal distribution. The Levene’s test was utilized to evaluate the homogeneity of variances, where *P*>.05 indicated homogeneous variances and vice versa. For statistical analysis, a two-way ANOVA was followed by Tukey’s post hoc test to analyze differences among groups. Behavioral scores were assessed using a repeated measures two-way ANOVA, with group and time as factors. Tukey’s post hoc test was then used to identify differences between groups. The statistical analysis was performed using SPSS 16, with *P*<.05 being considered statistically significant.

## Results

### The expression of the PKA/CREB signaling pathway was further enhanced in neurons after spinal cord injury by exercise therapy

**Figure 1:**
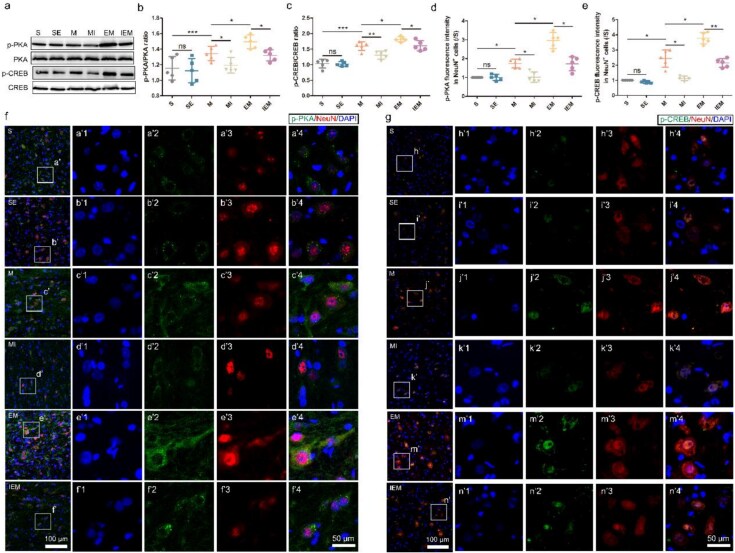
The expression of the PKA/CREB signaling pathway was further enhanced in neurons after spinal cord injury by exercise therapy. (**a**) Western blots and (**b-c**) quantification of p-PKA/PKA, p-CREB/CREB proteins in each group after SCI. The data are shown as means ± SD (*n* = 5). (**d**) Quantification of p-PKA fluorescence intensity in NeuN^+^ cell (/S). (**e**) Quantification of p-CREB fluorescence intensity in NeuN^+^ cell (/S). (**f**) Double staining (green: p-PKA; red: NeuN; blue: DAPI) and (**g**) (green: p-CREB; red: NeuN; blue: DAPI), scale bar: 50 μm & 100 μm. The data are shown as mean ± SD (*n* = 5). (^*^P<.05; ^**^P<.01; ^***^P<.001). *SE* sham-operated + ET, *M* SCI, *MI* SCI + H89, *EM* SCI + ET, *IEM* SCI + ET + H89

To confirm the effect of ET on the PKA/CREB signaling pathway, spinal cord tissue of the T9–T11 segments was measured by western blotting at 14 DPI (Figure 1a). As shown in Figure 1b and c, the expression of total PKA or CREB among the groups shows no difference (*P*>.05). No significant difference existed in the relative expression of p-PKA or p-CREB between groups SE and S (*P*>.05), but the expression was significantly higher after injury (p-PKA, M versus S: *P*<.001; p-CREB, M versus S: *P*<.001), and ET could further upregulate expression (p-PKA, *P*<.05; p-CREB, *P*<.05). In contrast, the inhibitor H89 can significantly downregulate the expression of p-PKA and p-CREB after SCI (*P*<.05). We performed co-staining of p-PKA/NeuN and p-CREB/NeuN to further confirm the phosphorylation of cytoplasmic PKA in neurons and the difference in the expression of phosphorylated CREB in nuclei among groups. Figure 1d and e depicts that p-PKA expression in the cytoplasm of neurons was upregulated after injury, especially after the ET intervention. Compared with group M, the inhibitor could effectively inhibit PKA phosphorylation (*P*<.001) (Figure 1f); p-CREB was mainly expressed in the nuclei of neurons, and the trends among groups were consistent with those for p-PKA (*P*<.001) (Figure 1g).

### Exercise therapy significantly improved the motor function of hindlimbs and promoted the formation of new neural circuits in rats through the PKA/CREB signaling pathway

**Figure 2:**
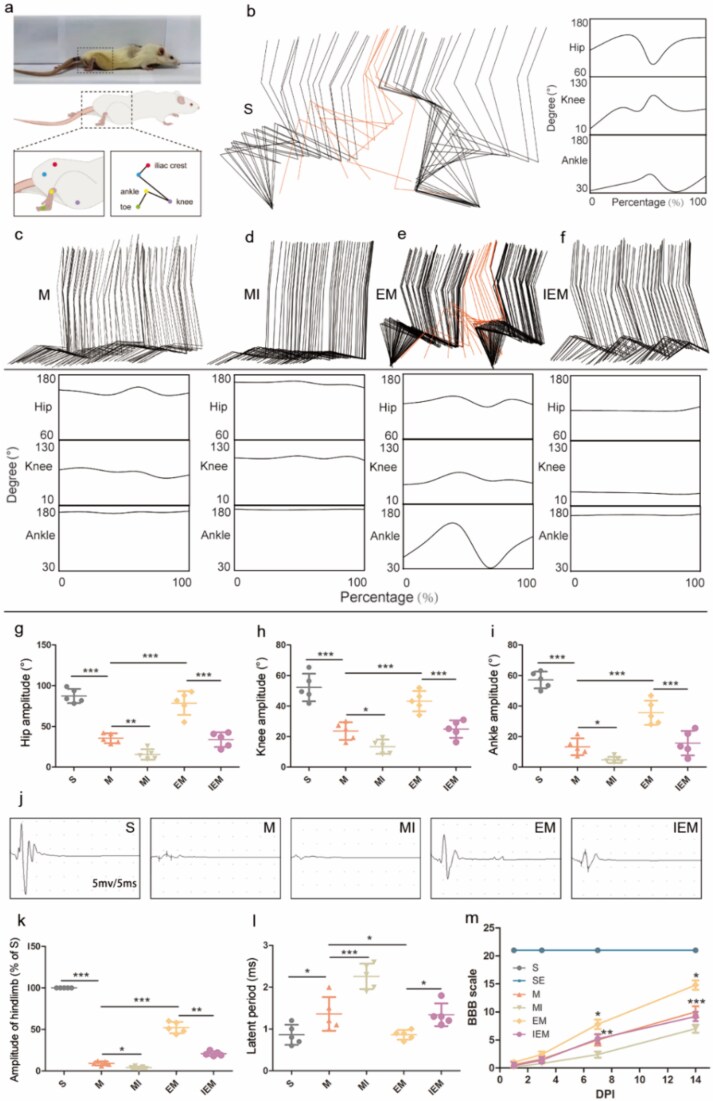
Exercise therapy significantly improved the motor function of hindlimbs and promoted the formation of new neural circuits in rats through the PKA/CREB signaling pathway. (**a**) Describes the schematic diagram of rats in the data collection of hindlimb motor joints. (**b-f**) Color-coded stick views illustrating hindlimb movements in representative rats from the S, SE, M, MI EM, and IEM groups. (**g-i**) Amplitude analysis of the hip angle (g), knee angle (h), and ankle angle (i) in the each group of rats; data are shown as means ± SD (*n* = 5). **(j**) Electrophysiological measurements of each group at 14 DPI. Each small space in the horizontal direction represents 5 ms, and each small space in the vertical direction represents 5 mV. (**k**) Quantitative analysis of average amplitudes relative to the sham group and (**l**) quantitative analysis of latent periods of each group. (**m**) BBB scale in the S, SE, M, MI EM, and IEM groups. Data are shown as means ± SD (*n* = 5). (^*^P<.05; ^**^P<.01; ^***^P<.001). *SE* sham-operated + ET, *M* SCI, *MI* SCI + H89, *EM* SCI + ET, *IEM* SCI + ET + H89

The results of the motion analysis of rat hind limbs (Figure 2a–i) revealed that the motor function of group EM was significantly better than that of group M, including greater angle oscillation of the hip, knee, and ankle joints (hip amplitude, EM versus M: *P*<.001; knee amplitude, EM versus M: *P*<.001; ankle amplitude, EM versus M: *P*<.001). Notably, the hip and knee joints were close to normal. Although the rats in groups MI and M displayed certain dragging behaviors, the joint angle in group MI was significantly lower than that in group M (hip amplitude, *P*<.01; knee amplitude, *P*<.05; ankle amplitude, *P*<.05). It is worth noting that the joint angle of group IEM was lower than that of group EM (hip amplitude, *P*<.001; knee amplitude, *P*<.001; ankle amplitude, *P*<.001), which was close to the level of group M. To thoroughly evaluate the effect of ET on the integration of damaged neurons into the injured site, we conducted an MEP experiment to monitor signal transduction ability. The results are shown in Figure 2j–l. In each intervention group, the latency from the spinal cord to the gastrocnemius muscle in group M was longer than that in group S after SCI, and the energy content of the impulse was significantly reduced (amplitude, *P*<.001; latent period, *P*<.05). The signal transduction ability of group EM was better than that of group M, and the latency was shorter (amplitude, *P*<.001; latent period, *P*<.05). In addition, the amplitude of the conduction signal in group MI was significantly lower than that in group M, and the latency was longer in group MI than that in group M (amplitude, *P*<.05, latent period, *P*<.001). In contrast, those in group IEM were smaller and longer than those in group EM (amplitude, *P*<.01, latent period, *P*<.05). The functional recovery underwent evaluation using BBB scale at 1, 3, 7, and 14 days after SCI. Based on experimental findings (Figure 2m), group EM exhibited markedly elevated BBB scores compared with group M at both 7 and 14 days (*P*<.05). Such findings strongly suggest that the enhanced motor function observed with ET may be regulated by the establishment of new circuit connections.

**Figure 3:**
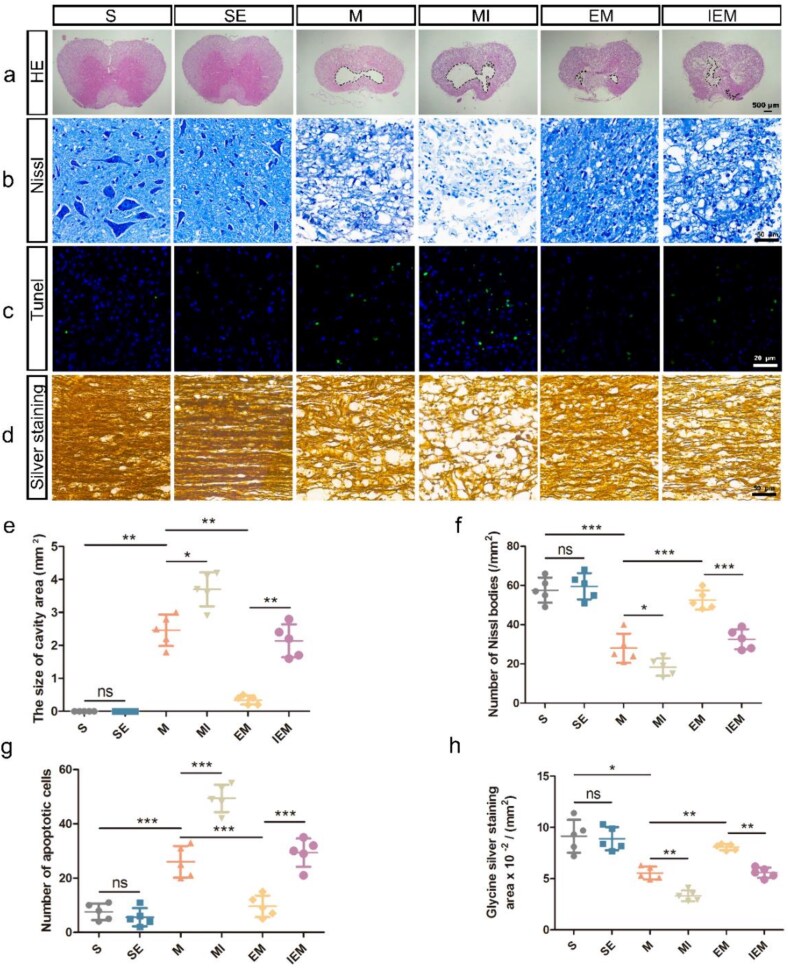
Exercise therapy significantly alleviated further tissue damage and apoptosis after SCI through the PKA/CREB signaling pathway and played a protective role in neurons. (**a**) HE-stained cross sections at 14 DPI, scale bar: 500 μm. The box formed by the dotted line in the picture indicates the area of the cavity. (**b**) Nissl staining in the S, SE, M, MI EM, and IEM groups, scale bar: 50 μm. (**c**) TUNEL staining showing the neuronal apoptosis in the epicenter, scale bar: 20 μm. (**d**) Glycine silver staining of nerve fibers of rats in each group, scale bar: 50 μm. (**e**) Quantification of the cavity area (mm^2^). (**f**) Quantification of the number of Nissl bodie (/mm^2^). (**g**) Quantification data of Tunel^+^ cells in each group. (**h**) Quantification of the glycine silver staining area in each group(/mm^2^). All data are shown as means ± SD (*n* = 5). (^*^P<.05; ^**^P<.01; ^***^P<.001). *SE* sham-operated + ET, *M* SCI, *MI* SCI + H89, *EM* SCI + ET, *IEM* SCI + ET + H89, *TUNEL* TdT-mediated dUTP nick end labeling

**Figure 4:**
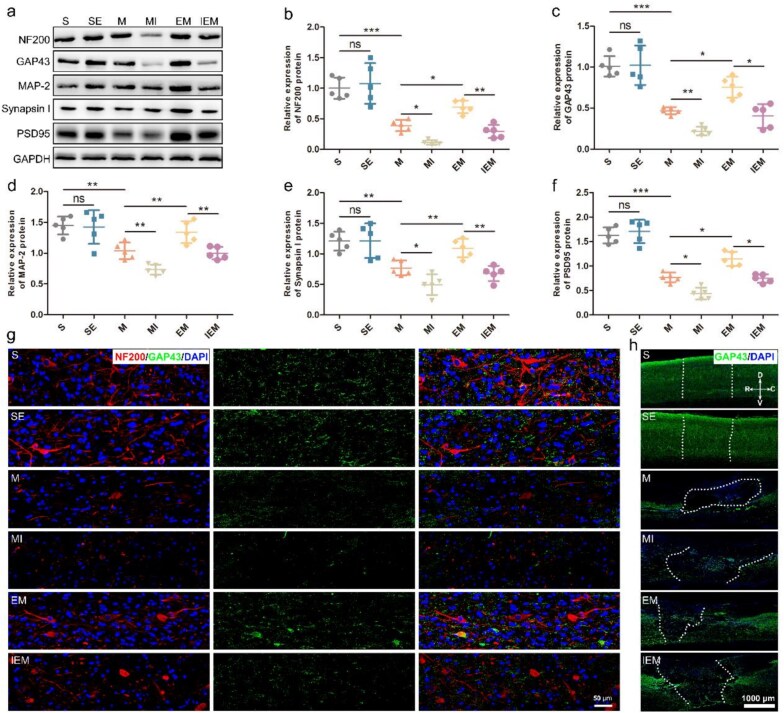
Exercise therapy enhanced the PKA/CREB signaling pathway closely related to dendritic/axonal/synaptic growth after SCI. (**a**) Western blots and (**b-f**) quantification of NF200, GAP43, MAP-2, Synapsin I, and PSD95 proteins in each group after SCI. The data are shown as means ± SD (*n* = 5). (^*^P<.05; ^**^P<.01; ^***^P<.001). (**g**) Double staining of sections in each group for GAP43 (green)/NF200 (red)/DAPI (blue), scale bar: 50 μm. (**h**) Staining of epicenter of spinal cord in each group for GAP43 (green) DAPI (blue), scale bar: 1000 μm. *SE* sham-operated + ET, *M* SCI, *MI* SCI + H89, *EM* SCI + ET, *IEM* SCI + ET + H89, *TUNEL* TdT-mediated dUTP nick end labeling, *D* dorsal, *V* ventral, *R* rostral, *C* caudal

**Figure 5:**
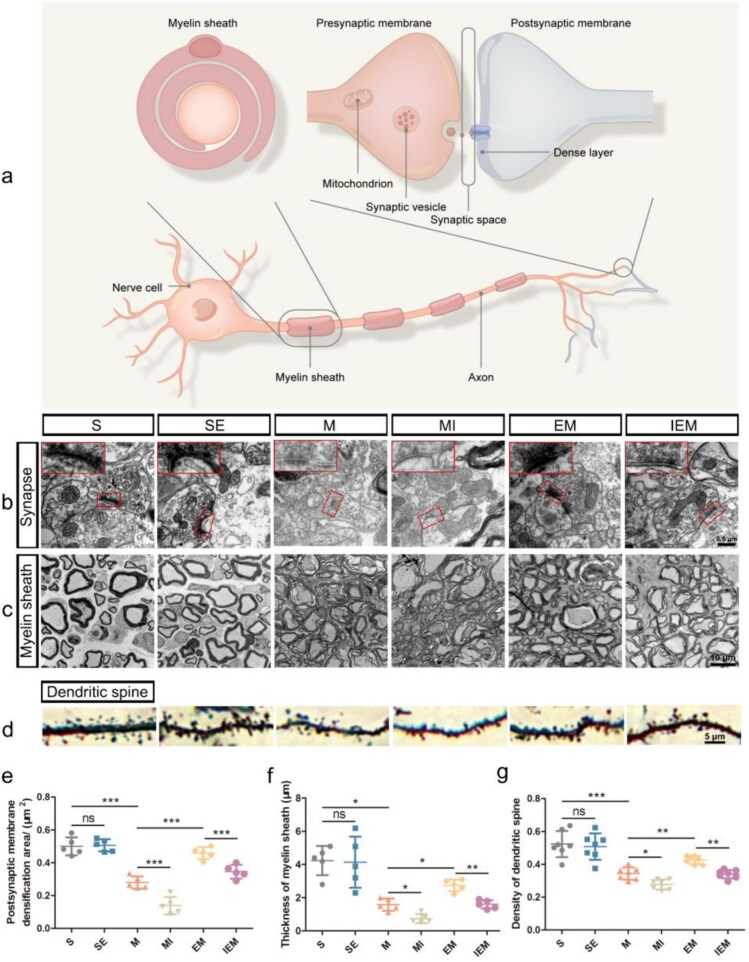
Exercise therapy enhanced the plasticity of synaptic/dendritic spines and myelin remodeling after SCI through the PKA/CREB signaling pathway. (**a**) Schematic diagram of neuron composition. (**b,c**) Transmission electron microscopy showed the synaptic structures and myelin sheath, and the scale bars indicated 0.5 μm (b) and10 μm (c). The red box in (b) shows the enlarged synaptic structure. (**d**) Examples of dendritic spines in each group, scale bar: 5 μm. (**e**) Quantification of the postsynaptic membrane densification area in each group. (**f**) Quantification of the thickness of myelin sheath in each group. (**g**) Quantification of the density of dendritic spine in each group. All data are shown as mean s± SD (*n* = 5). (^*^P<.05; ^**^P<.01; ^***^P<.001).*SE* sham-operated + ET, *M* SCI, *MI* SCI + H89, *EM* SCI + ET, *IEM* SCI + ET + H89

**Figure 6:**
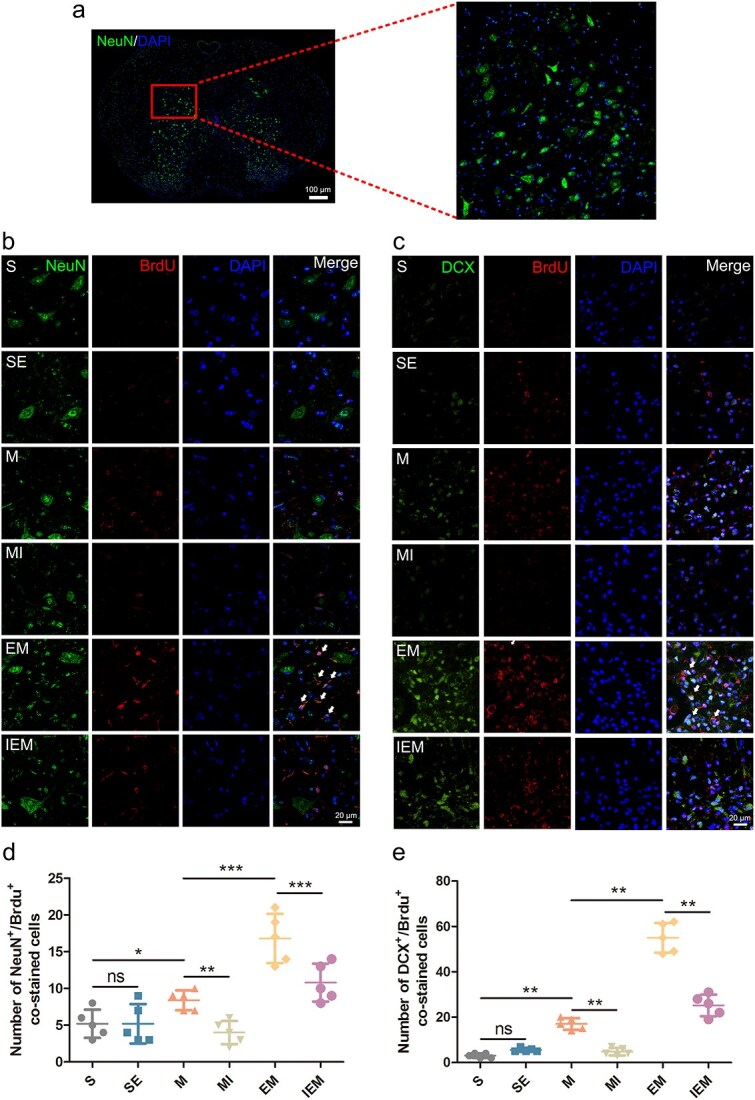
Exercise therapy promoted neuronal regeneration after SCI through the PKA/CREB signaling pathway. (**a**) Localization staining map of motor neurons in the IX lamina of the transverse section of the spinal cord, scale bar: 100 µm. (**b**) Double staining of sections from the spinal cord in each group of rats for NeuN (green)/BrdU (red)/DAPI (blue), scale bar: 20 μm. (**c**) Double staining (green: DCX; red: BrdU; blue: DAPI) from the spinal cord in each group of rats, scale bar: 20 μm. (**d**) Quantification of NeuN^+^/BrdU^+^ cells. (**e**) Quantification of DCX^+^/BrdU^+^ cells. All data are shown as means ± SD (*n* = 5) (^*^P<.05; ^**^P<.01; ^***^P<.001).*SE* sham-operated + ET, *M* SCI, *MI* SCI + H89, *EM* SCI + ET, *IEM* SCI + ET + H89

### Exercise therapy significantly alleviated further tissue damage and apoptosis after spinal cord injury through the PKA/CREB signaling pathway and played a protective role in neurons

To observe the destruction of tissue after SCI, crosscutting HE staining was performed at 14 DPI in the various groups (Figure 3a). The tissue morphology of groups S and SE was intact, and the structure was normal. However, after injury, we observed a significant tissue cavity in group M (*P*<.01). Blocking the PKA/CREB signaling pathway resulted in an even greater cavity area (MI versus M: *P*<.05, IEM versus EM: *P*<.01). After ET, the cavity area was significantly smaller (EM versus M: *P*<.01). Then, Nissl staining was used to detect nerve cell damage in each group (Figure 3b). Nissl bodies were large and numerous in groups S and SE, indicating that the protein synthesis function of nerve cells was stronger. However, no statistical significance existed between both groups (SE versus S: *P*>.05). Following SCI or using H89 led to a significant reduction in Nissl bodies (*P*<.001). Compared with group M, group EM showed a higher number of Nissl bodies (*P*<.001). We further assessed apoptosis in each group following SCI using TUNEL staining (Figure 3c). These results showed that apoptotic cell number in the hit center was significantly higher after injury (M versus S: *P*<.001). TUNEL^+^ cell number in group EM was markedly lower compared with group M (*P*<.001). When H89 was used, apoptotic cell number was significantly greater (*P*<.001). To observe the injury of nerve fibers in each group, glycine silver staining was used at 14 DPI (Figure 3d). The nerve fibers in groups S and SE were evenly distributed, regularly arranged, and normally shaped. After injury, the axons in group M were swollen, twisted, partially broken, and retracted (*P*<.05). Compared to group M, axonal damage in group MI was more serious (*P*<.01). After ET intervention, the situation improved significantly (*P*<.01). The above results show that ET plays an important role in neuronal protection (Figure 3e and f).

### Exercise therapy enhanced the PKA/CREB signaling pathway closely related to dendritic/axonal/synaptic growth after spinal cord injury

To understand the structural damage of neurons in different groups, we detected the typical proteins of dendrites, axons, and synapses using western blotting (Figure 4a). As the results show, NF200, GAP43, MAP2, Synapsin I, and PSD95 show similar expression trends in each group: the expression is significantly downregulated after SCI; the expression is further reduced after inhibitor intervention, while ET can be significantly increased the expression level (Figure 4b–f). To further show the axon growth at the injured site, we used double fluorescent staining of NF200 and GAP43. Following SCI, the positive area of GAP43^+^/NF200^+^cells was significantly lower. Contrarily, more GAP43 and NF200 expression was observed in group EM than in group M. After the use of the inhibitor H89, the fluorescent protein expression was significantly lower (Figure 4g). Besides, GAP43/DAPI staining results in longitudinal spinal cord sections suggested that, in the injured area, the growth of group ET was significantly higher than that of group M, while the growth of group IM was worse (Figure 4h).

### Exercise therapy promoted neuronal regeneration and enhanced the plasticity of synaptic/dendritic spines and myelin remodeling after SCI through the PKA/CREB signaling pathway

There were ultrastructural changes in neurons after SCI, including fragmentation of myelin sheaths and an increase in synaptic spaces (Figure 5a). To verify the roles of ET and H89, we used TEM to detect changes in synapses and myelin sheaths among groups (Figure 5b and c). The synaptic structures of groups S and SE were intact (*P*>.05). The number of synaptic vesicles and the area of postsynaptic density were significantly lower in group M (*P*<.001), while the synaptic membrane thickness was substantially uneven, a synaptic cleavage cavity was formed, and a synaptic connection could not be observed in group MI (*P*<.001). Finally, group EM improved compared to group M (*P*<.001), while group IEM showed more synaptic defects than group EM (*P*<.001). Golgi staining (Figure 5d) showed that the dendritic spine density of motor neurons after injury was significantly lower than that of group S (*P*<.001). Compared with group M, the density of dendritic spines was significantly higher in group EM (*P*<.01) and lower in group MI (*P*<.05). To verify whether ET could suppress damage to dendritic spines by upregulating PKA/CREB signaling pathway, the dendritic spine densities of groups IEM and EM were compared, indicating that they were significantly lower in group IEM (*P*<.01). Investigation of myelin sheaths showed that those of groups S and SE were arranged neatly and that the boundary was clear, but no statistically significant difference existed between both groups (*P*>.05). After injury, the thickness of myelin was significantly lower; it showed a loose and irregular state, its continuity was interrupted, and serious demyelination occurred (M versus S: *P*<.05). H89 aggravated demyelination after injury (MI versus M: *P*<.05). After the ET intervention, myelin sheaths were significantly remodeled, and their continuity and level of arrangement were relatively improved (EM versus M: *P*<.05). In comparison with group EM, the improvement of myelin in group IEM was significantly lower (*P*<.01) (Figure 5e–g). At 14 DPI, observing neuronal regeneration was achieved using the neuronal marker NeuN, newborn neuron marker DCX, and regeneration marker BrdU (Figure 6a–c). We took neurons from lamina IX of the spinal cord from each group for observation. We found that very few neurons regenerated in groups S and SE (*P*>.05); however, the number of regenerated neurons was greater following SCI (M versus S: *P*<.05). Interestingly, many regenerated neurons were observed in group EM (*P*<.001). The inhibitor H89 effectively inhibited the regeneration of neurons (Figure 6d and e).

## Discussion

In this study, we found that the PKA/CREB signaling pathway was related to myelin remodeling, dendritic spine remodeling, and spine plasticity of neurons. These structures were closely related to ET, which promoted the recovery of motor function after SCI. Our data also revealed a novel finding that ET could mediate the PKA/CREB signaling pathway in motoneurons in lamina IX of the spinal cord to promote nerve remodeling after SCI in rats.

The structure and function of neurons are linked, and the damage to neuron structure caused by injury is often accompanied by the loss of function [26]. After SCI, a large number of neurons die, and the axons, dendrites, and myelin sheath of neurons are destroyed. This makes it difficult to form an effective functional connection between neurons, resulting in impairment of motor function. Studies have shown that the reticulospinal tract can germinate below the lesion site after incomplete SCI [6]. Since we cannot replace dead motoneurons at present, it is an important goal for protecting motoneurons. Nerve remodeling is a powerful mechanism through which the CNS attempts to compensate for the structural or chemical destruction of functional connections between neurons [7]. It is well known that synapses exist mainly on dendrites and are the main determinants of neuronal integration and processing of afferent information; therefore, synaptic plasticity is critical in functional recovery. Maier and colleagues conducted a study showing that after unilateral corticospinal tract (CST) injury, forced induction of ET could promote axonal regeneration. Anterograde tracing has demonstrated that CST fibers can cross the midline and extend into the contralateral denervated gray matter, leading to functional recovery [5, 27]. Behavioral results of our study indicated that ET could promote the recovery of motor function after SCI, which suggests that neural circuit remodeling occurred in rats. The comparison of groups S, M, and EM also ruled out differences in self-recovery of rats. Furthermore, our staining results showed obvious apoptosis, accompanied by less neuronal activity after SCI in rats. However, we were surprised to find that there were significantly more newborn neurons in the penumbra in group EM, accompanied by less apoptosis. One aspect of neural plasticity is the enhancement and weakening of synaptic responses to inputs. Under normal and pathological conditions, such synaptic changes are crucial for processes such as learning, memory, and motor output [28–30]. Changes in dendrites and synapses are the foundation for recovering the motor function. In this study, ET was employed to attenuate SCI-induced dendrite atrophy as well as synaptic damage. ET protected dendrites and synapses, enabling them to keep connection patterns and take part in the neural network of the spinal cord. Following the trend of recovery of motor function, ET promoted motor recovery by inhibiting dendritic loss of motoneurons and promoting myelin remodeling and synaptic plasticity after SCI.

Plasticity is an extraordinary characteristic of the CNS, enabling it to learn and recover from insults. However, without rehabilitation training, functional improvement after SCI is limited. The effects of many rehabilitation interventions on functional recovery related to anatomical and physiological changes in the spinal cord have been studied [8, 31]. A study conducted by Pahul’s team showed that exercise dependence increased the regulation of axonal regeneration of intrinsic spinal cord neurons and regeneration-related genes after SCI [32]. Odpchi *et al*. believe that long-term forced treadmill exercise is more beneficial to the spinal cord than to the brain in mice [33]. An interesting study also suggests that treadmill exercise promotes functional recovery in rats with SCI and that mechanical epigenetic changes in the motor cortex may contribute to the improvement of exercise induction [34]. Hydrostatic pressure plays an important role in eliminating limb swelling, improving vital capacity, and improving physical endurance. The physical stimulation of water on the surface of the skin can improve blood circulation, promote muscle relaxation, reduce spasms and pain, increase the painless range of motion, and improve balance and motor function. Given the fact that it is difficult for paralyzed lower limbs to move after SCI, we made the rats undergo active rehabilitation training under water flow and a conveyor belt [17]. A comparison of groups EM and M revealed that the expression of p-PKA and p-CREB levels in neurons was higher after the ET intervention. This suggests that the PKA/CREB pathway can be further enhanced by ET. Histological staining showed that ET could also significantly improve the pathological conditions of the cavity, reduction in the number of Nissl bodies, apoptosis, and nerve fiber osteoporosis caused by injury. Interestingly, in the double staining of BrdU/NeuN and BrdU/DCX, we also found more regenerated neurons in group EM. Furthermore, the expression levels of NF200, GAP43, MAP-2, synapsin I, and PSD95 were significantly higher in group EM, which was consistent with increased number of dendritic spines and the repair of synaptic plasticity. This series of results reveals that ET plays an indispensable role in promoting recovery from SCI.

CNS injury can activate the PKA/CREB signal transduction pathway as an endogenous protective mechanism [35, 36]. The phosphorylation of PKA activates the phosphorylation site Ser133 of the downstream CREB, starts the transcription of downstream genes, and plays a protective role on damaged nerve cells. Research has shown that stimulating the PKA/CREB signaling pathway can promote the secretion of a variety of neurotrophic factors, which is beneficial to nerve remodeling [37, 38]. Some researchers have also found that phosphorylation of CREB increases the excitability of hippocampal and striatal neurons in rodents, while inhibition reduces the excitability of neurons [39]. Reports indicate that CREB plays a role in embryonic neuronal development and PNS regeneration, and may be involved in the development and regeneration of the adult CNS [12, 40]. Following SCI, pathological mechanisms and signaling pathways involved exhibit complexity. Our findings revealed that ET resulted in significantly higher expression of p-PKA and p-CREB compared to group M. Such findings may be correlated with delayed necrotic or apoptotic death of CREB-producing cells in spinal anterior neurons following SCI, with ET contributing to the survival of these cells. As expected, we observed that p-PKA was mainly expressed in the cytoplasm of neurons, while p-CREB was expressed in the nucleus. This is consistent with the signaling pathway that has been elucidated, indicating that the phosphorylation of PKA induced by the ET intervention further promoted the phosphorylation of CREB in the nucleus and ultimately promoted nerve remodeling.

To further understand the function of PKA/CREB signaling pathway in ET-mediated nerve remodeling, we injected intrathecal H89, a widely used and highly selective PKA/CREB inhibitor, for comparison. We found that the expression of p-PKA and p-CREB in neurons decreased after the use of H89, suggesting that H89 has an excellent pathway inhibition effect. When the PKA/CREB signaling pathway was blocked, the tissue damage was aggravated and the neuronal activity decreased in group MI, accompanied by mixed nerve fibers and a large number of apoptotic cells. This partly explains why the group MI tended to have worse behavioral scores. The expression of recurrent neuronal and neural structural markers in the MI group was significantly lower than that in the M group. Unsurprisingly, after the use of H89, the myelin damage of neurons, loss of dendritic spines, and synaptic destruction were aggravated. According to such results, we can reasonably hypothesize that PKA/CREB signaling pathway may directly promote nerve remodeling by modulating neurogenesis. We subsequently investigated the association between exercise-induced nerve remodeling and PKA/CREB signaling pathway, and we compared groups EM and IEM. The behavioral scores of rats in the EM group were significantly higher than those in the IEM group, suggesting that the PKA/CREB signaling pathway was involved in the ET-mediated recovery of motor function. Interestingly, the improvement of the cavity area, neuronal activity, and nerve fibers induced by ET was reversed by the inhibitor H89, which further suggests that part of the tissue improvement mediated by ET was achieved through the PKA/CREB signaling pathway. Given our results and those of various other in-depth studies, we have finally determined that ET-induced neuronal regeneration and neuronal structural protection are inseparable from the PKA/CREB signaling pathway.

To sum up, this study’s findings demonstrated that the neuroprotective effect of ET was mediated in part by regulating PKA/CREB signaling pathway to promote nerve remodeling after SCI. Nevertheless, acknowledging major limitations of this research should be considered. First, confirmatory experiments often cannot identify specific mechanisms. With our study, we could not rule out the effects of other signaling molecules caused by different ETs on the experimental results. Second, in clinical practice, gender differences often produce different results of diseases and interventions. With our experimental design, we could not verify a gender difference. Finally, because of limited experimental conditions, we failed to measure the neuroelectrophysiology of the unicellular after the intervention and could not assess membrane signaling and changes in the excitability of single cells. Given the deficiency of the experimental design, we will strive to improve it in future experiments.

## Conclusions

To summarize, our findings indicated the following. (1) ET counteracted the H89-induced suppression of the PKA/CREB signaling pathway following SCI; (2) ET significantly alleviated neuronal injury and improved motor dysfunction; (3) ET facilitated neuronal regeneration by mediating the PKA/CREB signaling pathway; (4) ET enhanced synaptic and dendritic spine plasticity, as well as myelin sheath remodeling, post-SCI through the PKA/CREB signaling pathway.

## Data Availability

The data that support the findings of this study are available from the corresponding author upon reasonable request.
